# MAVS phosphorylation acts as a cellular stress sensor that modulates antiviral immunity

**DOI:** 10.1016/j.isci.2025.113256

**Published:** 2025-07-31

**Authors:** Dongyi Zhao, Nao Morimoto, Riho Saito, Juri Yamada, Shuntaro Abe, Hidetaka Kosako, Yukiko Gotoh, Tomohiko Okazaki

**Affiliations:** 1 Laboratory of Molecular Cell Biology, Institute for Genetic Medicine, Hokkaido University, Sapporo, Hokkaido 060-0815, Japan; 2 Graduate School of Pharmaceutical Sciences, The University of Tokyo, Tokyo 113-0033, Japan; 3 Institute of Advanced Medical Sciences, Tokushima University, Tokushima 770-8503, Japan; 4 International Research Center for Neurointelligence (WPI-IRCN), The University of Tokyo, Tokyo 113-0033, Japan; 5 Japan Science and Technology Agency (JST) Fusion Oriented Research for Disruptive Science and Technology (FOREST) Program, Kawaguchi 332-0012, Japan

**Keywords:** Biochemistry, Immune response, Cell biology

## Abstract

Upon viral infection, cytosolic RIG-I-like receptors recognize viral RNA and activate innate immune responses through the mitochondrial antiviral-signaling protein (MAVS), leading to type I interferon (IFN) production and apoptosis. Cellular stress influences immune activation, but its impact on MAVS signaling remains largely unclear. Here, we show that MAVS undergoes phosphorylation via p38 MAPK signaling, activated by the stress-activated MAPKKK ASK1. This modification enhances MAVS interaction with TRAF, a key downstream adaptor, thereby promoting type I IFN induction. Oxidative and endoplasmic reticulum stress significantly amplified type I IFN expression upon viral infection, but this effect was attenuated in cells expressing MAVS mutants lacking phosphorylation sites. These findings suggest MAVS phosphorylation as a key mechanism integrating cellular stress signals into antiviral immunity. By linking the MAPK pathway to MAVS-dependent IFN expression, we propose MAVS phosphorylation as a cellular stress sensor that modulates antiviral immunity in a context-dependent manner.

## Highlights

• MAVS is phosphorylated by stress-responsive ASK1–p38 MAPK signaling
• MAVS phosphorylation enhances type I IFN induction via recruitment of key downstream adaptors
• Phosphorylation selectively amplifies IFN expression but not apoptosis upon viral infection
• Cellular stress enhances MAVS signaling via phosphorylation to fine-tune antiviral immunity

## Graphical abstract

**Figure:**
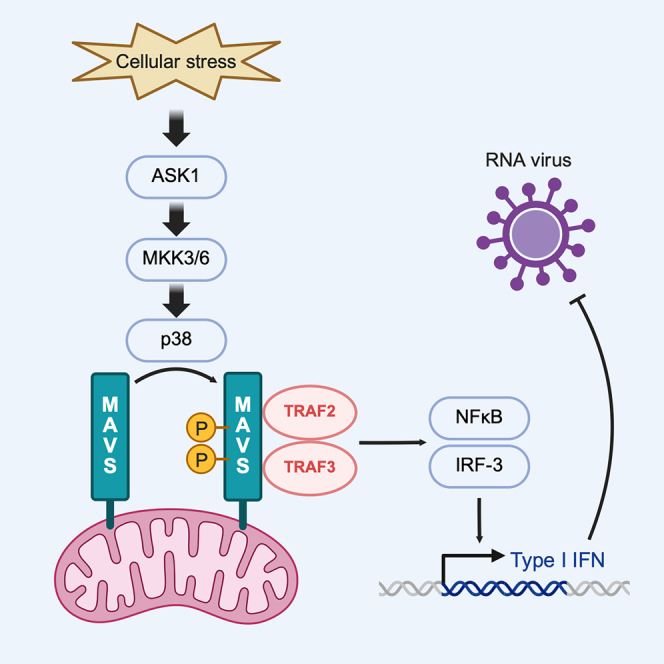

## Introduction

Cells constantly encounter a variety of stressors, including oxidative stress and endoplasmic reticulum (ER) stress. Initially characterized as mechanisms that restore cellular homeostasis, cellular stress responses are now recognized as pivotal modulators of immune responses, either directly or indirectly, through interactions with immune signaling pathways.^1^ Through this regulation, cellular stresses not only influence the quantitative aspects, but also the qualitative nature of immune responses, enabling adaptive tuning of immunity under stress conditions.^2^^,^^3^ This adaptive modulation allows the host to effectively recover from these potentially life-threating and complex situations.

In response to viral infections, host cells deploy first-line defense mechanisms such as apoptosis and type I interferon (IFN) production to reduce the viral load and maintain homeostasis.^4^^,^^5^^,^^6^^,^^7^ RIG-I-like receptors (RLRs), a subfamily of pattern recognition receptors that detect pathogen-associated molecular patterns, recognize viral RNA in the cytosol and interact with the mitochondrial antiviral-signaling protein (MAVS, also known as IPS-1, VISA, or CARDIF).^8^^,^^9^^,^^10^^,^^11^^,^^12^^,^^13^^,^^14^ MAVS acts as a central adaptor that links viral recognition to downstream signaling pathways such as the interferon regulatory factor 3 (IRF3) pathway, the nuclear factor-kappa B (NF-κB) pathway, and the mitogen-activated protein kinase (MAPK) p38 and c-Jun N-terminal kinase (JNK) pathways, through direct interactions with TNF-associated factor (TRAF) family proteins.^15^^,^^16^^,^^17^^,^^18^^,^^19^^,^^20^ Together, these pathways orchestrate transcription of type I IFN genes and execution of apoptosis, contributing to robust antiviral responses.^21^^,^^22^^,^^23^^,^^24^^,^^25^^,^^26^

Among these pathways, the MAPK p38 and JNK pathways have the potential to integrate cellular stress signals into antiviral immune responses.^27^ These pathways are regulated by upstream MAPK kinases (MAPKKs) MKK3/6 and MKK4/7, respectively, as well as (further upstream) MAPK kinase kinases (MAPKKKs), including the apoptosis signal-regulating kinase (ASK) family.^28^^,^^29^ The ASK family is implicated in MAVS-dependent type I IFN production and apoptotic signaling during viral infections.^16^ Furthermore, ASK family members are activated by diverse cellular stressors, including oxidative stress and ER stress, positioning them as potential mediators of crosstalk between stress conditions and MAVS signaling.^29^^,^^30^^,^^31^^,^^32^ Indeed, oxidative stress enhances production of type I IFN via MAVS,^33^ and ASK family members modulate the balance between MAVS-mediated apoptosis and IFN induction.^16^ These findings suggest the possibility that cellular stress not only amplifies the strength of antiviral responses but also dictates their specific outcomes. Despite these insights, the mechanisms by which stress pathways regulate MAVS activity and immune responses remain largely unexplored.

Here, we describe a novel mechanism that links cellular stress to MAVS-dependent immune responses during viral infection. We identify ASK1 as a key mediator of MAVS phosphorylation at specific S186 and S220 residues via activation of the p38 MAPK pathway, which is distinct from the previously reported TBK1-mediated phosphorylation at the C-terminal serine cluster.^34^ This ASK1–p38–dependent phosphorylation supports interaction between MAVS and key downstream adaptors (i.e., TRAFs) and promotes efficient activation of the NF-κB and IRF-3 pathways. Notably, MAVS phosphorylation selectively enhances type I IFN induction, but not induction of apoptosis, effectively balancing these antiviral responses. Furthermore, we demonstrate that cellular stressors, including oxidative stress and ER stress, amplify MAVS-mediated immune responses through this phosphorylation. Our findings highlight the intricate interplay between cellular stress pathways and innate immune signaling, providing critical insight into how stress conditions shape antiviral immunity.

## Results

### ASK1-MKK6-p38 axis induces phosphorylation of MAVS at S^186^ and S^220^

**Figure 1:**
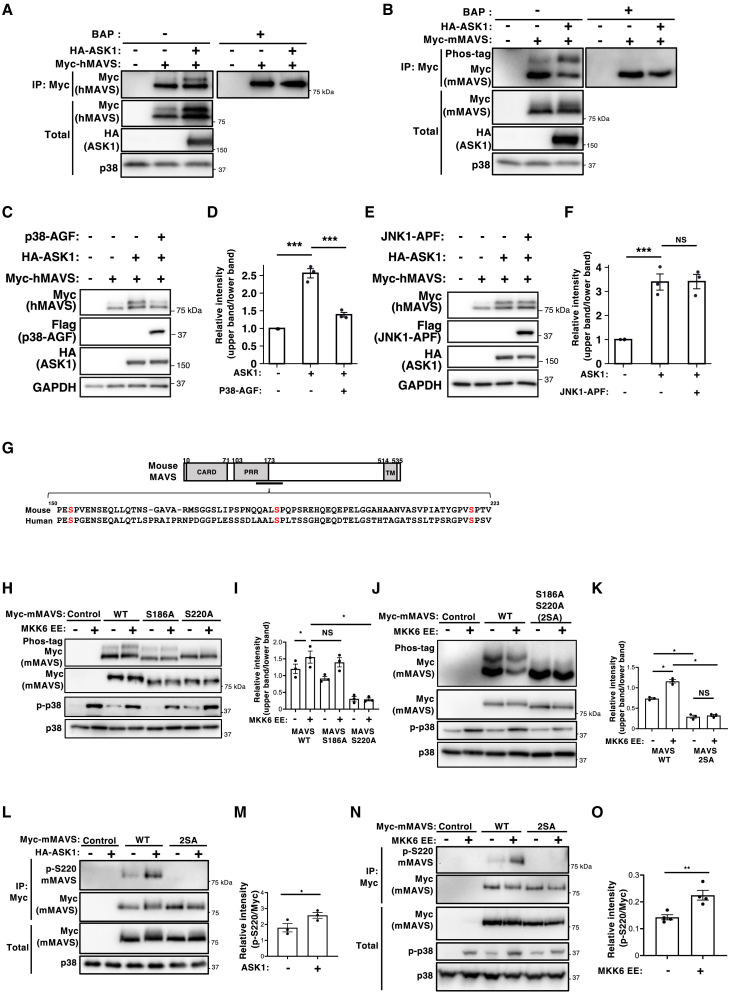
The ASK1-MKK6-p38 axis induces phosphorylation of MAVS at S^186^ and S^220^ (A) HEK293T cells transiently transfected for 20 h with expression vectors for HA-tagged ASK1 and Myc-tagged human MAVS were subjected to immunoprecipitation (IP) with an anti-Myc antibody. The resulting precipitates were treated with bacterial alkaline phosphatase (BAP). The precipitates and original cell lysates (Total) were then immunoblotted with antibodies specific for Myc, p38, and ASK1. Results are representative of three independent experiments. (B) HEK293T cells transiently transfected for 20 h with expression vectors for HA-tagged ASK1 and Myc-tagged mouse MAVS were subjected to IP with an anti-Myc antibody. The resulting precipitates were treated with BAP and (along with the original cell lysates (Total)) separated by SDS-PAGE in Phos-tag gels or normal gels, respectively, followed by immunoblot analysis with antibodies specific for Myc, p38, or ASK1. Results are representative of three independent experiments. (C–F) HEK293T cells were transiently transfected for 20 h with expression vectors for HA-tagged ASK1, Myc-tagged WT hMAVS, Myc-tagged hMAVS 2SA, Flag-tagged p38 AGF (C, D), or Flag-tagged JNK1 APF (E, F). The cell lysates were then separated by SDS-PAGE, and then subjected to immunoblot analysis with antibodies indicated. Results are representative of three independent experiments. Densitometry analysis of upper band relative to the lower band generated by anti-Myc antibodies in the immunoblot. Data are expressed as the mean ± SEM from three independent experiments (∗∗∗*p* < 0.001, not-significant (NS), one-way ANOVA with Tukey’s multiple comparison test). (G) Domain structure of hMAVS. Three serine residues (S^152^, S^186^, and S^220^), all of which are conserved between humans and mice and located immediately before proline residues, are indicated in red. CARD, caspase activation and recruitment domain; PRR, proline rich region; TM, transmembrane. (H and I) HEK293T cells were transiently transfected for 20 h with expression vectors for MKK6 EE and Myc-tagged WT mMAVS, Myc-tagged mMAVS S186A, or S220A. The cell lysates were then separated by SDS-PAGE in Phos-tag gels or normal gels, and then subjected to immunoblot analysis with antibodies specific for Myc, phospho-p38 (p-p38), or p38 (H). Results are representative of three independent experiments. Densitometry analysis of the upper band relative to the lower band generated by anti-Myc antibodies in the immunoblot of the Phos-tag gel (I). Data are expressed as the mean ± SEM from three independent experiments (∗*p* < 0.05, non-significant (NS), one-way ANOVA with Dunnett's multiple comparison test). (J and K) HEK293T cells were transiently transfected for 20 h with expression vectors for MKK6 EE and Myc-tagged WT mMAVS or Myc-tagged mMAVS S186A S220A (2SA). The cell lysates were separated by SDS-PAGE in Phos-tag gels or normal gels and then subjected to immunoblot analysis with antibodies specific for Myc, p-p38, or p38 (J). Results are representative of three independent experiments. Densitometry analysis of the upper band relative to the lower band generated by anti-Myc antibodies in the immunoblot of the Phos-tag gel (K). Data are expressed as the mean ± SEM from three independent experiments (∗*p* < 0.05, non-significant (NS), one-way ANOVA with Tukey’s multiple comparisons test). (L and M) HEK293T cells transiently transfected for 20 h with expression vectors for HA-ASK1 and Myc-tagged WT mMAVS, or Myc-tagged mMAVS S186A/S220A (2SA) were subjected to IP with an anti-Myc antibody, and the resulting precipitates were then subjected to immunoblot analysis, together with the original cell lysates (Total), with antibodies specific for phospho-S^220^ MAVS, Myc, or p38 (L). Results are representative of three independent experiments. Densitometry analysis of the phospho-S^220^ MAVS band relative to the myc band after IP (M). Data are expressed as the mean ± SEM from three independent experiments (∗*p* < 0.05, two-tailed Student’s *t* test). (N and O) HEK293T cells transiently transfected for 20 h with expression vectors for MKK6 EE and Myc-tagged WT mMAVS, or Myc-tagged mMAVS S186A/S220A (2SA) were subjected to IP with an anti-Myc antibody. The resulting precipitates were then subjected to immunoblot analysis, together with the original cell lysates (Total), with antibodies specific for phospho-S^220^ MAVS, Myc, p-p38, or p38 (N). Results are representative of three independent experiments. Densitometry analysis of the phospho-S^220^ MAVS band relative to the Myc band after IP (O). Data are expressed as the mean ± SEM from three independent experiments (∗∗*p* < 0.01, two-tailed Student’s *t* test).

Previously, we showed that ASK1 mediates expression of type I IFN through the RLR-MAVS pathway,^16^ although the underlying mechanisms remain unclear. While investigating downstream targets of the ASK1-MAPK pathway within the RLR-MAVS signaling cascade, we observed that overexpression of ASK1 enhances a band shift of human MAVS (hMAVS) and mouse MAVS (mMAVS) in immunoblot analyses (Figures 1A and 1B). This band shift was likely attributable to phosphorylation, as it was abolished by treatment of immunoprecipitated MAVS with phosphatase. Moreover, analysis of Phos-tag gels, which retard migration of phosphorylated proteins, revealed an enhanced band shift of mMAVS when ASK1 was expressed (Figure 1B). Collectively, these findings suggest that ASK1 promotes phosphorylation of both human and mouse MAVS. Since ASK1 functions as an upstream MAPKKK of MAPKs p38 and JNK, we investigated whether phosphorylation of MAVS is mediated by these MAPKs. Phos-tag immunoblotting revealed that the p38 inhibitor SB202190 significantly reduced the ASK1-induced band shift of mMAVS, whereas the JNK inhibitor SP600125 had little effect (Figures S1A and S1B). Furthermore, expression of a dominant-negative form (AGF) of p38 markedly suppressed the ASK1-induced phosphorylation shift of hMAVS, whereas JNK1 APF had no apparent effect (Figures 1C–1F). These results suggest that p38, but not JNK, plays a pivotal role in ASK1-induced phosphorylation of MAVS. To identify the phosphorylation sites, we overexpressed and isolated His-hMAVS, generated tryptic peptides, and subjected them to liquid chromatography-tandem mass spectrometry (LC-MS/MS) analysis. Although we identified several phosphorylation sites, we focused on S^152^, S^186^, and S^220^ because these residues are conserved in both humans and mice, and all are positioned immediately before a proline, a motif typical of proline-directed kinases such as p38 MAPK (Figure 1G; Table S1). Phos-tag immunoblotting revealed that the S152A mutation did not affect the band shift of MAVS induced by constitutively active MKK6 (MKK6EE), an MAPKK of p38 (Figure S2A). However, the S186A mutation partially reduced the band shift induced by MKK6EE, whereas the S220A mutation and the combined S186A/S220A (2SA) mutation abolished it almost completely (Figures 1H–1K). This suggests that both S^186^ and S^220^ are phosphorylated through the MKK6-p38 axis. To further investigate MAVS phosphorylation, we generated an antibody specific for the phosphorylated S^220^ residue. The raised antibody recognized WT MAVS but failed to detect MAVS mutants harboring an alanine substitution at the phosphorylation site (Figure 1L). Notably, the intensity of the signal obtained by the anti-phospho-S^220^ antibody increased upon overexpression of either ASK1 or MKK6EE (Figures 1L–1O), further supporting the notion that MAVS is phosphorylated downstream of the ASK1-p38 signaling pathway. Importantly, this ASK1-induced phosphorylation shift was still observed upon TBK1 knockdown (Figures S3A and S3B), suggesting that this occurs independently of TBK1. Moreover, the phosphorylation shift was also seen in the MAVS 2SA mutant upon TBK1 overexpression (Figure S4A), consistent with previous reports that TBK1 phosphorylates a C-terminal serine cluster including S442. These findings collectively indicate that the ASK1–p38 axis directly regulates MAVS phosphorylation at S186 and S220.

### MAVS phosphorylation sites are important for type I IFN expression, but not for caspase activation, in response to poly(I:C) stimulation

**Figure 2:**
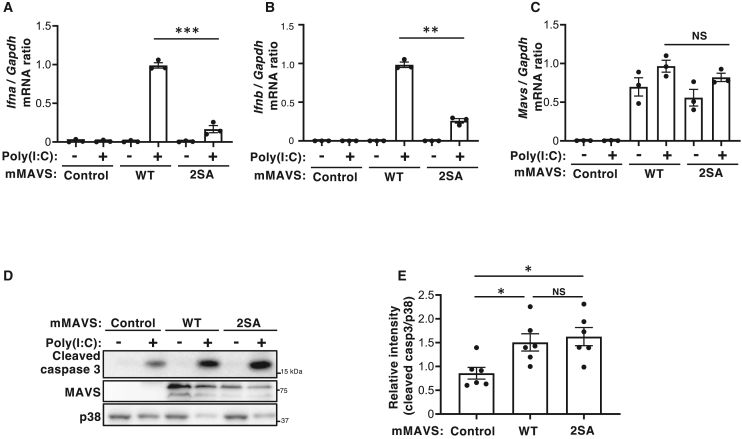
MAVS phosphorylation sites are important for expression of type I IFNs, but not for caspase activation, in response to poly(I:C) stimulation (A–C) MEFs isolated from MAVS KO mice were reconstituted with plasmids encoding either WT or the S186A/S220A (2SA) mutant form of mMAVS and transfected with 0.25 μg/mL poly(I:C) for 3 h. Next, levels of *Ifna* (A), *Ifnb1* (B), or *Mavs* (C) mRNA were measured by reverse transcription and quantitative polymerase chain reaction (RT-qPCR). Data are presented as the mean ± SEM of three independent experiments (∗∗*p* < 0.01, ∗∗∗*p* < 0.001, non-significant (NS), two-tailed Student’s *t* test). (D and E) MEFs isolated from MAVS KO mice were reconstituted with plasmids encoding either the WT or 2SA mutant form of mMAVS, and then transfected with 0.25 μg/mL poly(I:C) for 12 h. Cells were then subjected to immunoblot analysis with antibodies specific for cleaved caspase 3, MAVS, or p38 (D). Data are representative of six independent experiments. Densitometry analysis of the cleaved caspase 3 band relative to the p38 band (E). Data are expressed as the mean ± SEM of six independent experiments (∗*p* < 0.05, non-significant (NS), one-way ANOVA with Tukey’s multiple comparisons test).

To investigate the role of MAVS phosphorylation, we next examined how mutations at the phosphorylation sites affect IFN expression, proinflammatory cytokine expression, and caspase activation in response to cytoplasmic double-stranded RNA (dsRNA). Using MAVS-knockout (KO) mouse embryonic fibroblasts (MEFs), we established cells stably expressing either wildtype (WT) MAVS or the phosphorylation-deficient 2SA mutant, and then assessed their responses to polyinosinic-polycytidylic acid (poly(I:C)), a synthetic dsRNA analog. WT MAVS-expressing cells showed robust induction of type I IFNs (IFNα and IFNβ) following transfection with poly(I:C). By contrast, this induction was markedly attenuated in cells expressing the MAVS 2SA mutant (Figures 2A–2C). Similar results were obtained for the induction of the proinflammatory cytokines Il-6 and Tnfα (Figures S5A and S5B). These findings suggest that phosphorylation of MAVS is required for effective induction of type I IFNs and proinflammatory cytokines in response to dsRNA. By contrast, poly(I:C)-induced activation of caspases was comparable between WT MAVS- and MAVS 2SA mutant-expressing cells (Figures 2D and 2E), suggesting that phosphorylation of MAVS is dispensable for induction of apoptosis. We also found that MAVS 2SA mutation did not affect its interaction with NLRP3 (Figure S6A). These results indicate that while phosphorylation of MAVS selectively enhances the induction of type I IFN and proinflammatory cytokines, it does not contribute to NLRP3 inflammasome activation or apoptosis induction.

### MAVS phosphorylation sites facilitate activation of downstream signaling and interactions between MAVS and TRAF2/3

**Figure 3:**
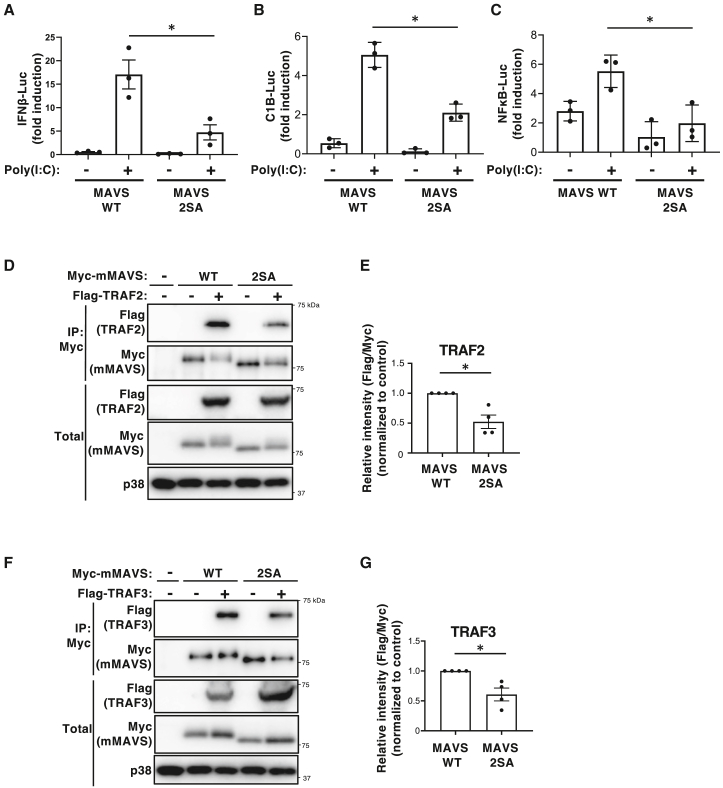
MAVS phosphorylation sites facilitate activation of downstream signaling, and interaction between MAVS and TRAF2/3 (A–C) MAVS KO MEFs reconstituted with MAVS WT or the S186A/S220A (2SA) mutant were transiently transfected with a Renilla luciferase reporter plasmid under the control of the mouse *Ifnb1* promoter (IFN-β-Luc) (A), repeated IRF-binding sites (C1B-Luc) (B), or NF-κB-binding sites (NF-κB-Luc) (C), along with Firefly luciferase expression plasmids (control) in the presence of 0.25 μg/mL poly(I:C)). The cells were then lysed and Luc activity was measured. Activity of *Renilla* Luc was normalized by that of Firefly Luc. Data are expressed as the mean ± SEM of three independent experiments (∗*p* < 0.05, two-tailed Student’s *t* test). (D and E) HEK293T cells transiently transfected for 20 h with expression vectors for Flag-tagged TRAF2 and Myc-tagged mMAVS were subjected to immunoprecipitation (IP) with an anti-Myc antibody. The resulting precipitates were then subjected to immunoblot analysis, together with the original cell lysates (Total), with antibodies specific for Flag, Myc, or p38 (D). Results are representative of four independent experiments. Densitometry analysis of the Flag band relative to the Myc band in the immunoprecipitate (E). Data are expressed as the mean ± SEM from four independent experiments (∗*p* < 0.05, one-sample *t-*test). (F, G) HEK293T cells transiently transfected for 20 with expression vectors for Flag-tagged TRAF3 and Myc-tagged mMAVS were subjected to IP with and anti-Myc antibody. The resulting precipitates were then subjected to immunoblot analysis, together with the original cell lysates (Total), with antibodies specific for Flag, Myc, or p38 (F). Results are representative of four independent experiments. Densitometry analysis of the Flag band relative to the Myc band in the immunoprecipitate (G). Data are expressed as the mean ± SEM from four independent experiments (∗*p* < 0.05, one-sample *t-*test).

To explore how phosphorylation of MAVS enhances IFN induction, we first considered the possibility that it promotes interaction with upstream RIG-I–like receptors (RLRs). However, co-immunoprecipitation experiments using MDA5 revealed that binding was not decreased in the MAVS 2SA mutant but rather increased (Figures S7A and S7B), suggesting that phosphorylation does not enhance MAVS–RLR interactions. We then examined activation of transcription factors IRF-3 and NF-κB, both of which are key mediators of type I IFN expression. We found that whereas poly(I:C) increased the activity of a reporter gene construct containing the IFN-β promoter, as well as those containing IRF-binding sites or NF-κB-binding sites, in WT MAVS-expressing cells, these activities were significantly impaired in MAVS 2SA mutant-expressing cells (Figures 3A–3C). These findings suggest that phosphorylation of MAVS amplifies activation of the IRF-3 and NF-κB pathways. Since TRAF family proteins mediate MAVS-dependent activation of these pathways through direct interactions,^11^^,^^12^^,^^18^^,^^19^^,^^20^^,^^35^^,^^36^ we next investigated whether phosphorylation of MAVS affects the MAVS-TRAF interaction. Co-immunoprecipitation analysis showed that the interaction between the MAVS 2SA mutant and TRAF2/TRAF3 was significantly weaker than that between MAVS WT and TRAF2/TRAF3 (Figures 3D–3G). We also examined the association between MAVS WT and TRAF5 or TRAF6, but were unable to do so in our system. These data suggest that phosphorylation of MAVS facilitates interaction with key downstream adaptors, at least TRAF2 and TRAF3, while potential interactions with TRAF5 and TRAF6 remain to be investigated.

### MAVS phosphorylation sites are important for induction of type I IFNs and defense against RNA viruses

**Figure 4:**
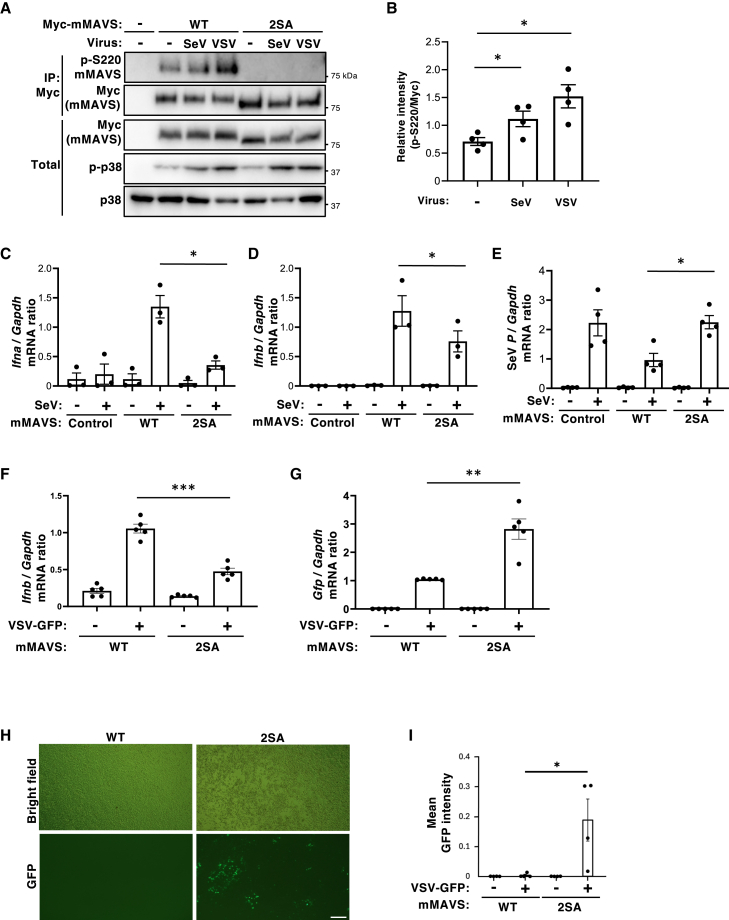
Phosphorylation of MAVS is important for type I IFN induction and for antiviral defense against infection by RNA viruses (A and B) HEK293T cells transiently transfected for 20 h with expression vectors for Myc-tagged WT mMAVS or Myc-tagged mMAVS S186A/S220A (2SA) were infected with Sendai virus (SeV) or Vesicular Stomatitis Virus (VSV), and then subjected to immunoprecipitation (IP) with an anti-Myc antibody. The resulting precipitates were then subjected to immunoblot analysis, together with the original cell lysates (Total), with antibodies specific for phospho-S^220^ MAVS, Myc, p-p38, or p38 (A). Results are representative of four independent experiments. Densitometry analysis of the phospho-S^220^ MAVS band relative to the Myc band in the immunoprecipitate (B). Data are expressed as the mean ± SEM from four independent experiments (∗*p* < 0.05, two-tailed Student’s *t* test). (C–E) MAVS KO MEFs reconstituted with MAVS WT or the 2SA mutant were infected with SeV for 3 h. Next, levels of *Ifna* (C), *Ifnb1* (D), or Sendai virus *P* (E) mRNA were measured by reverse transcription and quantitative polymerase chain reaction (RT-qPCR) analysis. Data are expressed as the mean ± SEM of four independent experiments (∗*p* < 0.05, two-tailed Student’s *t* test). (F and G) MAVS KO MEFs reconstituted with MAVS WT or the 2SA mutant were infected with VSV-GFP for 3 h. Next, levels of *Ifnb1* (F), and *Gfp* (G) mRNA were measured by reverse transcription and quantitative polymerase chain reaction (RT-qPCR) analysis. Data are expressed as the mean ± SEM of five independent experiments (∗∗*p* < 0.01, ∗∗∗*p* < 0.001, two-tailed Student’s *t* test). (H and I) MAVS KO MEFs reconstituted with MAVS WT or the 2SA mutant were infected with VSV-GFP, and GFP fluorescence was analyzed 24 h post-infection. Representative fluorescence images were shown. Scale bars, 250 μm (H). Quantification of mean GFP intensity per field (I). Data represent mean ± SEM from four independent experiments (∗*p* < 0.05; one-way ANOVA with Tukey’s multiple comparisons test).

Next, we investigated whether phosphorylation of MAVS plays any role in defense against RNA virus infection. First, we examined phosphorylation of MAVS upon infection with RNA viruses such as Sendai virus (SeV) or vesicular stomatitis virus expressing green fluorescent protein (VSV-GFP). Immunoblot analysis revealed that signals detected by the MAVS p-S^220^ antibody were significantly enhanced in response to infection with either virus, indicating a potential role for MAVS phosphorylation in antiviral responses (Figures 4A and 4B). Notably, this virus-induced phosphorylation was significantly attenuated in ASK1 knockout cells, suggesting that ASK1 is required for robust MAVS phosphorylation during viral infection (Figures S8A and S8B). Furthermore, whereas infection of MAVS WT cells with SeV or VSV-GFP induced type I IFNs, this response was significantly suppressed in cells expressing the phosphorylation-deficient MAVS 2SA mutant. (Figures 4C, 4D, and 4F). Similar reductions were also observed for proinflammatory cytokines Il-6 and Tnfα following viral infection, indicating that MAVS phosphorylation contributes broadly to antiviral cytokine responses (Figures S9A–S9D). Consistent with these results, the levels of SeV and VSV-GFP RNA were significantly higher in cells expressing MAVS 2SA than in cells expressing MAVS WT (Figures 4E and G), and GFP fluorescence following VSV-GFP infection was also markedly elevated in MAVS 2SA-expressing cells (Figures 4H and 4I). These findings suggest that phosphorylation of MAVS enhances the induction of type I IFNs in response to viral infection, and plays a critical role in antiviral defense.

### Cellular stresses enhance type I IFN expression via MAVS phosphorylation sites

**Figure 5:**
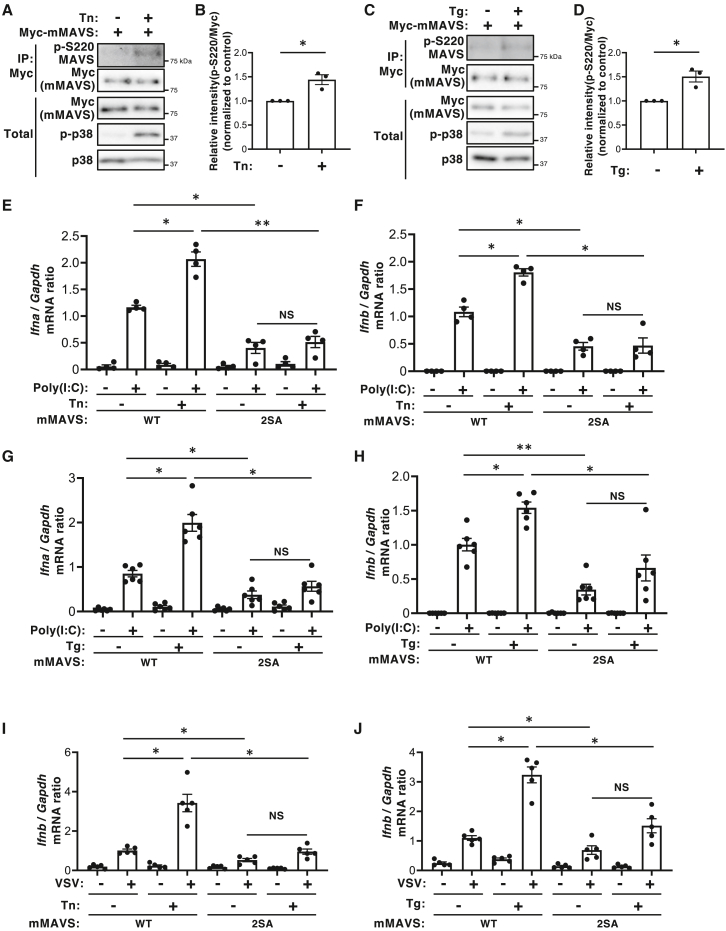
ER stress increases expression of type I IFNs via phosphorylation of MAVS (A and B) HEK293T cells transiently transfected for 20 h with expression vectors for Myc-tagged WT mMAVS were treated with 100 μg/mL of tunicamycin (Tn) for 3 h. The lysates were then subjected to immunoprecipitation (IP) with and anti-Myc antibody. The resulting precipitates were subjected to immunoblot analysis, together with the original cell lysates (Total), with antibodies specific for phospho-S^220^ MAVS, Myc, p-p38, or p38 (A). Results are representative of three independent experiments. Densitometry analysis of the phospho-S^220^ MAVS band relative to the Myc band in the immunoprecipitate (B). Data are expressed as the mean ± SEM from three independent experiments (∗*p* < 0.05, one-sample *t-*test). (C and D) HEK293T cells transiently transfected for 20 h with expression vectors for Myc-tagged WT mMAVS were treated with 3 μM of thapsigargin (Tg) for 3h. The lysates were then subjected to IP with an ant-Myc antibody. The resulting precipitates were then subjected to immunoblot analysis, together with the original cell lysates (Total), with antibodies specific for phospho-S^220^ MAVS, Myc, p-p38, or p38 (C). Results are representative of three independent experiments. Densitometry analysis of the phospho-S^220^ MAVS band relative to the Myc band in the immunoprecipitate (D). Data are expressed as the mean ± SEM from three independent experiments (∗*p* < 0.05, one-sample *t-*test). (E and F) MAVS KO MEFs were reconstituted with MAVS WT or the 2SA mutant and treated with 100 μg/mL of Tn for 3h. Cells were then transfected for 3 h with 0.25 μg/mL poly(I:C) and subjected to reverse transcription and quantitative polymerase chain reaction (RT-qPCR) analysis to detect *Ifna* (E) or *Ifnb1* (F) mRNA. Data are expressed as the mean ± SEM of four independent experiments (∗*p* < 0.05, ∗∗*p* < 0.01, non-significant (NS), one-way ANOVA with Tukey’s multiple comparisons test). (G and H) MAVS KO MEFs were reconstituted with MAVS WT or the 2SA mutants and treated for 3 h with 3 μM of Tg. Cells were then transfected with 0.25 μg/mL poly(I:C) for 3 h, and subjected to reverse transcription and quantitative polymerase chain reaction (RT-qPCR) analysis to detect *Ifna* (G) or *Ifnb1* (H) mRNA. Data are expressed as the mean ± SEM of six independent experiments (∗*p* < 0.05, ∗∗*p* < 0.01, non-significant (NS), one-way ANOVA with Tukey’s multiple comparisons test). (I and J) MAVS KO MEFs were reconstituted with MAVS WT or the 2SA mutant treated for 3 h with 100 μg/mL of Tn (I) or 3 μM of Tg (J). cells were then infected with VSV for 3 h, and subjected to reverse transcription and quantitative polymerase chain reaction (RT-qPCR) analysis to detect *Ifnb1* mRNA. Data are expressed as the mean ± SEM of five independent experiments (∗*p* < 0.05, non-significant (NS), one-way ANOVA with Tukey’s multiple comparisons test).

Given that ASK1 and its downstream effector p38 are activated by various cellular stressors including ER stress and oxidative stress,^29^^,^^30^^,^^31^^,^^32^ and that MAVS phosphorylation sites are necessary for activation of the IRF-3 and NF-κB pathways (Figures 3B and 3C), we hypothesized that these stresses might modulate antiviral responses via ASK1-p38-induced MAVS phosphorylation and the subsequent activation of the IRF-3 and NF-κB pathways. To test this, we first treated cells with the ER stress inducers tunicamycin (Tn) or thapsigargin (Tg). We found that treatment with either Tn or Tg significantly enhanced the signal detected by the MAVS p-S^220^ antibody (Figures 5A–5D), suggesting that ER stress enhances phosphorylation of MAVS. Next, we investigated whether ER stress modulates antiviral responses. Expressions of type I IFNs upon dsRNA stimulation significantly increased in cells treated with Tn or Tg (Figures 5E–5H). Importantly, this increase in type I IFN transcription induced by ER stress inducers was not observed in cells expressing the MAVS 2SA mutant (Figures 5E–5H). These data suggest that ER stress enhances type I IFN expression in response to dsRNA stimulation via MAVS phosphorylation. Furthermore, we examined the level of type I IFN expression upon VSV infection, and found again that ER stress inducers increased expression of IFN-β after RNA virus infection in a MAVS phosphorylation-dependent manner (Figures 5I and 5J). We then examined the effect of oxidative stress using rotenone, a mitochondrial complex I inhibitor that induces reactive oxygen species (ROS) production. Consistent with a previous study,^33^ rotenone treatment increased expression of type I IFN in response to dsRNA stimulation (Figures S10A and S10B). Importantly, the effect of rotenone was dependent on MAVS phosphorylation sites (Figures S10A and S10B). Moreover, this dependency on phosphorylation sites was also observed after infection with SeV or VSV-GFP (Figures S10C–S10E). Collectively, these findings suggest that cellular stressors such as ER stress and oxidative stress enhance MAVS phosphorylation, which likely amplifies activation of the IRF-3 and NF-κB pathways, ultimately contributing to increased production of type I IFNs.

## Discussion

Our study uncovers a previously uncharacterized mechanism by which cellular stress pathways regulate MAVS-dependent antiviral immune responses. Specifically, we demonstrate that the ASK1-p38 MAPK axis induces phosphorylation of MAVS at S^186^ and S^220^, which are conserved across species. This phosphorylation is essential for robust induction of type I IFNs in response to infection with RNA viruses, but not for MAVS-mediated apoptotic signaling, suggesting a specific role for phosphorylation in tailoring antiviral innate immunity.

Previously, we proposed that ASK1 homo-oligomers selectively promote IFN production via the p38 pathway, while ASK1-ASK2 hetero-oligomers preferentially induce apoptosis through the JNK pathway, during viral infection^16^; however, the mechanisms underlying these selective MAVS downstream responses remained unclear. Our finding that activation of the ASK1-p38 pathway increases MAVS phosphorylation to drive IFN induction without affecting caspase activity highlights the selective role of the p38 pathway during IFN induction. We also found that JNK phosphorylates MAVS at sites distinct from S^186^ and S^220^ (unpublished observation), raising the intriguing possibility that these modifications selectively contribute to apoptotic signaling. Future studies to identify these JNK-mediated phosphorylation sites and to elucidate how distinct phosphorylation events modulate MAVS interactions and functional outcomes will provide insight into the bifurcation between IFN induction and apoptosis.

Our findings also suggest that phosphorylation of MAVS serves as a critical node in a positive feedback mechanism that amplifies MAVS-dependent production of type I IFN. Previously, we demonstrated that ASK1 is activated downstream of MAVS,^16^ now positioning ASK1 as both an effector and a regulator of MAVS signaling. This MAVS-ASK1 feedback loop may ensure rapid and sustained induction of type I IFNs during viral infection. Particularly during the early stages of infection, this mechanism enables the host to mount an effective antiviral response while preserving tissue integrity by prioritizing induction of IFN over apoptotic signaling.

Beyond its role in antiviral signaling, phosphorylation of MAVS via the ASK1-p38 pathway serves to integrate cellular stress signals into immune responses. We demonstrate that cellular stresses promote phosphorylation of MAVS, which in turn enhance production of type I IFN in response to dsRNA stimulation, as well as RNA virus infection. Considering that ER stress and oxidative stress are often associated with progression of viral replication,^37^^,^^38^ this integration of stress signals may fine-tune antiviral responses so that they are proportionate to the extent of viral replication. Furthermore, ASK1 is activated not only by oxidative stress and ER stress, but also by various other stressors such as osmotic stress and inflammatory cytokines,^29^ raising an intriguing possibility that ASK1 serves as a critical mediator that links diverse stress pathways to MAVS signaling. During activation of such stress pathways, activation of ASK1 may amplify phosphorylation of MAVS and subsequent production of IFN, thereby allowing cells to adjust their immune responses to the changing environmental conditions.^1^^,^^39^

Our findings suggest that phosphorylation of MAVS may also be influenced by metabolic signals, providing an additional layer of immune regulation. By reanalyzing phosphoproteomics datasets derived from insulin-stimulated cells reported by other studies,^40^^,^^41^ we identified increased phosphorylation of MAVS at S^186^ and S^220^. Combined with our data showing that phosphorylation at these sites increases expression of type I IFNs, this raises the intriguing possibility that insulin signaling regulates MAVS activity via the ASK1-p38 MAPK pathway, as insulin is also known to activate both ASK1 and p38.^42^^,^^43^ Importantly, in cases of advanced insulin resistance or type 2 diabetes, conditions characterized by disrupted insulin signaling or impaired sensitivity, phosphorylation of MAVS and subsequent induction of IFN may be compromised, potentially contributing to the increased susceptibility of individuals with metabolic disorders to infection with RNA viruses such as influenza and SARS-CoV-2.^44^ Further studies are required to clarify the functional impact of MAVS phosphorylation under this metabolically stressed environment.

In conclusion, our study identifies MAVS as an integrative hub connecting viral recognition with stress signaling pathways to promote robust and context-specific production of IFNs. These findings highlight MAVS phosphorylation as a potential target for modulating immune responses during viral infection and pathophysiological stress conditions. Further exploration of MAVS phosphorylation under diverse contexts will provide new opportunities for therapeutic intervention.

### Limitations of the study

While our study identifies S186 and S220 as critical MAVS phosphorylation sites mediated by the ASK1–p38 axis, the potential roles of other phosphorylation sites —such as those potentially targeted by the JNK pathway— remain to be investigated. Additionally, interactions between MAVS and TRAF5 or TRAF6 could not be detected under our experimental conditions, possibly due to technical limitations. Finally, although our findings provide strong support for stress-regulated MAVS phosphorylation in cell-based systems, *in vivo* validation of these mechanisms will be essential to fully understand their physiological relevance.

## Resource availability

### Lead contact

Further information and requests for resources and reagents should be directed to and will be fulfilled by the lead contact, Tomohiko Okazaki (okazaki@igm.hokudai.ac.jp).

### Materials availability

The genetically manipulated cells and datasets generated during and/or analyzed during the current study are available from the corresponding authors on reasonable request.

### Data and code availability

All data used in this manuscript can be found in the main paper and supplementary materials. The mass spectrometry proteomics data have been deposited to the ProteomeXchange Consortium via the jPOST partner repository with the dataset identifier **PXD064922**, and are available at the following URL: https://repository.jpostdb.org/entry/JPST003870.0.

## Acknowledgments

We thank Fumi Yamamoto, Noriko Kato, and Ayaka Abe for technical assistance; Seiichi Sato and Akinori Takaoka for providing the NLRP3 construct. This research was partly supported by the Promotion Project for Young Investigators in 10.13039/501100005946Hokkaido University, and Medical Research Center Initiative for High Depth Omics.

This study was supported by grants from the following funding sources.

The 10.13039/501100001700Ministry of Education, Culture, Sports, Science and Technology of Japan (MEXT) [JP16K19149 (T.O.), JP18K07168 (T.O.), JP21K07064 (T.O.), JP24K02172 (T.O.), JP24K22007 (T.O.), JP15H05773 (Y.G.), JP16H06481 (Y.G.), JP16H06479 (Y.G.), JP16H06279 (Y.G.), JP22H00431 (Y.G.), JP22H04925 (PAGS) (Y.G.), JP24H02322 (Y.G.), JP18gm0610013 (Y.G.), JP24gm1310004 (Y.G.)]; The FOREST Program of the Japan Science and Technology Agency (JPMJFR204Q, T.O.); The Takano Life Science Research Foundation (T.O.); The Chemo-Sero-Therapeutic Research Institute (T.O.); The Mitsubishi Foundation (T.O.); The Naito Foundation (T.O.); The Secom Foundation (T.O.); The Astellas Foundation for Research on Metabolic Disorders (T.O.); and the Nagase Science and Technology Foundation (Y.G.).

## Author contributions

D.Z. performed the majority of the experiments and wrote the initial draft of the manuscript; N.M., R.S., J.Y., and S.A. performed experiments and wrote the manuscript; H.K. performed LC-MS/MS analysis and analyzed the results; Y.G. supervised the project; T.O. supervised the project and wrote the manuscript.

## Declaration of interests

The authors declare no competing interests.

## STAR★Methods

### Key resources table

**Table:** 

REAGENT or RESOURCE	SOURCE	IDENTIFIER
**Antibodies**		

Anti-phosphoS220 MAVS Antibody	Eurofins	acetyl-TYGPV(phospho)SPTVS
anti-Myc (9E10)	Santa Cruz Technology	sc-40; RRID:AB_627268
anti-HA(Y-11)	Santa Cruz Technology	sc-805; RRID:AB_631618
anti-p38(c-20)	Santa Cruz Technology	sc-535; RRID:AB_632138
anti–phospho-p38 (Thr180/Tyr182)	Cell Signaling	9211; RRID:AB_331641
anti–cleaved caspase-3 (D175) 5A1E	Cell Signaling	9664; RRID:AB_2070042
anti-MAVS (rodent specific)	Cell Signaling	4983; RRID:AB_823566
anti–phospho-IRF3 (Ser396) 4D4G	Cell Signaling	4947; RRID:AB_823547
anti-TBK1/NAK (D1B4)	Cell Signaling	3504; RRID:AB_2255663
rabbit polyclonal anti-Flag	Rockland Inc.	600-401-383; RRID:AB_219374
Amersham, ECL Rabbit IgG, HRP-linked whole Ab (from donkey)	Cytiva	NA934; RRID:AB_772206
Amersham, ECL Mouse IgG, HRP-linked whole Ab (from sheep)	Cytiva	NA931; RRID:AB_772210

**Bacterial and virus strains**		

Sendai virus	American Type Culture Collection	Cantell strain, VR-907
Vesicular stomatitis virus-green fluorescent protein (VSV-GFP)	This paper	

**Chemicals, peptides, and recombinant proteins**		

SB202190	Med Chem Express	HY-10295
SP600125	Sigma Aldrich	S5567-10 MG
Rotenone	Sigma Aldrich	R8875-1G
Thapsigargin	Tocris	1138
Tunicamycin	LKT Labs, Inc.	T8153
RNAiso	TaKaRa	9108
Dulbecco’s Modified Eagle’s Medium (DMEM)	Sigma Aldrich	D6429
Fetal bovine serum	Gibco	10437028
Penicillin-streptomycin	Nacalai Tesque Inc	06168–34
Lipofectamine Transfection Reagent	Invitrogen	18324020
Poly(I:C)	GE Healthcare	11545784
Lipofectamine 2000	Invitrogen	11668019
GeneJuice	Merck Millipore	70967
Phos-stop	Roche	4906845001
Calf Intestinal Alkaline Phosphatase	Takara	
Puromycin	Sigma Aldrich	P-8833

**Critical commercial assays**		

ReverTra Ace™ qPCR RT Master Mix	Toyobo	FSQ301
KAPA SYBR® FAST qPCR Master Mix	NIPPON Genetics Co. Ltd	KK4602
Dual-Luciferase® Reporter Assay System	Promega	E1910

**Deposited data**		

Proteomic identification of phosphorylation sites of MAVS	This study	https://repository.jpostdb.org/entry/JPST003870.0 PXD064922

**Experimental models: cell lines**		

*Mavs*^−/−^ MEFs	S. Akira (Osaka University)	
HEK-293T	Y. Gotoh (The University of Tokyo)	
*Mavs* WT MEFs	This paper	
*Mavs* 2SA MEFs	This paper	
ASK1^−/−^ HEK-293T	This paper	
BHK/T7-9 cells	RIKEN BRC	RCB4942

**Oligonucleotides**		

Gapdh forward 5’-ATGAATACGGCTACAGCAACAGG-3′	Okazaki et al.^16^	
Gapdh reverse 5’-CTCTTGCTCAGTGTCCTTGCTG-3′	Okazaki et al.^16^	
Mouse Ifnα forward 5’-CCTGTGTGATGCAGGAACC-3′	Okazaki et al.^16^	
Mouse Ifnα reverse 5’-TCACCTCCCAGGCACAGA-3′	Okazaki et al.^16^	
Mouse Ifnβ forward 5’-CCTGTGTGATGCAGGAACC-3′	Okazaki et al.^16^	
Mouse Ifnβ reverse 5’-CTTTGCACCCTCCAGTAATAGC-3′	Okazaki et al.^16^	
Sendai virus P gene forward 5′-GCATGGAGCCTGGCAGCTCA-3′	Okazaki et al.^16^	
Sendai virus P gene reverse 5’-CGATTCAGCGGTGGGGACCG-3′	Okazaki et al.^16^	
VSV-GFP forward 5’-GAACGGCATCAAGGTGAACT-3’	This paper	
VSV-GFP reverse 5’-TGCTCAGGTAGTGGTTGTCG-3’	This paper	
Mouse IL6 forward 5’-TGCCTTCATTTATCCCTTGAA-3′	This paper	
Mouse IL6 reverse 5’-TTACTACATTCAGCCAAAAAGCA-3′	This paper	
Mouse TNFα forward 5’-TGGCCTCCCTCTCATCAGTT-3′	This paper	
Mouse TNFα reverse 5’-GCTTGTGACTCGAATTTTGAGAAG-3′	This paper	
Mouse *Mavs* S220A sense primer 5’-CCTATGGACCTGTGGCTC CAACCGTTTCC-3′	This paper	
Mouse *Mavs* S220A anti-sense primer 5’-GGAAACGGTTGGAGCCAC AGGTCCATAGG-3′	This paper	
ASK1 gRNA forward 5’-CACCGTGCCCCTGG CATCGGTTGTC-3’	This paper	
ASK1 gRNA reverse 5’-AAACGACAACCGATG CCAGGGGCAC-3’	This paper	

**Recombinant DNA**		

PiggyBac-CAG-MAVS WT/2SA-IRES-puro	This paper	
pCAG-hyPBase	VectorBuilder	
pPB-CAG-empty-pgk-hph	Addgene	#48753
pSpCas9-GFP	Addgene	#79144
PB-IRES-puro vector	Funakoshi	PB210PA-1
pRK5-Flag TRAF2	H. Ichijo (Institute of Science Tokyo)	
pRK5-Flag TRAF3	H. Ichijo (Institute of Science Tokyo)	
pcDNA3-HA-ASK1	H. Ichijo (Institute of Science Tokyo)	
pcDNA3-MKK6 EE	Y. Gotoh (The University of Tokyo)	
pCXN Flag mTBK1	Y. Gotoh (The University of Tokyo)	
pcDNA3.1 Flag NLRP3	A. Takaoka (Hokkaido University)	
pcDNA3 Flag p38 AGF	This paper	
pcDNA3 Flag JNK1β1 APF	Y. Gotoh (The University of Tokyo)	
pcDNA3-Myc-mouse*Mavs* WT	This paper	
pcDNA3-Myc-mouse*Mavs* S186A	This paper	
pcDNA3-Myc-mouse*Mavs* S220A	This paper	
pcDNA3-Myc-mouse*Mavs* S186AS220A	This paper	
IFNβ (p125-RLuc)	T Fujita (Kyoto University)	
IRF3 (p55C1B RLuc)	T Fujita (Kyoto University)	
pEF BOS Flag-hMDA5-N	T Fujita (Kyoto University)	
NF-κB (pNF-κB-RLuc)	This paper	
pGL3-control	Promega	

**Software and algorithms**		

LightCycler 480 system	Roche	
Prism 8	Graphpad	
ImageJ	Fiji	

### Experimental model and study participant details

#### Viruses

Sendai virus (Cantell strain, VR-907) was purchased from the American Type Culture Collection. The plasmid encoding vesicular stomatitis virus-green fluorescent protein (VSV-GFP) was constructed by inserting the P2A and GFP sequences at the 3’ end of G protein coding sequence into pVSV-FL+(2) (Kerafast, EH1002). The helper plasmids (pBS-IRES-N-φT/pBS-IRES-L-φT/pBS-IRES-P-φT) was generated by inserting IRES elements into the Kpn I site of pBS-N-φT, pBS-L-φT, pBS-P-φT, respectively. VSV-GFP was recovered by transfecting pVSV-FL+(2) and the helper plasmids into BHK/T7-9 cells (RIKEN RBC, RCB4942) using polyethylenimine (Polysciences Inc., 24765).

#### Generation of ASK1 KO cells

ASK1 KO 293T cells were generated as previously described.^45^ For construction of the CRISPR vector for human *MAP3K5* deletion, pairs of oligonucleotides encoding the guide RNA (gRNA) were designed, annealed, and ligated into the px458 vector.^46^ The sequences used were as follows: human *MAP3K5*, 5'-CACCGTGCCCCTGGCATCGGTTGTC-3' and 5'- AAACGACAACCGATGCCAGGGGCAC-3'.

### Method details

#### Cell culture and transfection

MAVS KO MEFs were kindly provided by Dr. S Akira (Osaka University). HEK293T cells and MEFs were cultured in Dulbecco’s Modified Eagle’s Medium (DMEM; Sigma Aldrich, D6429) supplemented with 10% fetal bovine serum (Gibco, 10437028) and 100 U/mL penicillin-streptomycin (Nacalai Tesque Inc., 06168-34). Stable cell lines expressing MAVS WT or MAVS S186A/S220A (2SA) were generated by co-transfecting 0.5 μg of PB-CAG-MAVS WT/2SA-IRES-puro and 0.5 μg of pCAG-hyPBase into 1 × 10^5^ MAVS KO MEFs using the Lipofectamine Transfection Reagent (Invitrogen, 18324020). Transfected cells were selected using puromycin (1 μg/mL; Sigma Aldrich, P-8833). Polyinosinic-polycytidylic acid (poly(I:C)) was purchased from GE Healthcare (11545784) and transfected into cells at 0.25 μg/mL using Lipofectamine 2000 (Invitrogen, 11668019).

#### Quantification of GFP fluorescence following VSV-GFP infection

MAVS KO MEFs reconstituted with MAVS WT or 2SA mutants were seeded at a density of 3.5×10^5^ cells per well in a 12-well plate. They were infected with VSV-GFP (3.5x10^4^ PFU/well) and GFP intensity was measured after 24 h. Bright field and GFP fluorescent images were obtained with Zeiss AX10 and Axiocam 208 color. Quantification was performed using ImageJ (version 2.1.0; National Institutes of Health, Bethesda, MD). Images were first converted to 8-bit grayscale, and background was subtracted by setting minimum value for “brightness and contrast” as 40. and mean intensity was taken using the “Measure” function. This process was applied uniformly to all images. Data were exported to excel for statistical analysis. A minimum of 3 fields per condition were analyzed from at least 4 independent experiments.

#### RNA interference

The Silencer Select siRNA system (ThermoFisher Scientific) was used for knockdown of TBK1 in HEK293T cells. Cells were transfected with TBK1 siRNA (# 4427038, ID: s761) or Negative Control #2 siRNA (# 4390846) using Lipofectamine RNAi MAX reagent (Thermo Fisher Scientific) according to the manufacturer's instructions. The transfection was performed for 4 days before further analysis. The TBK1 siRNA sequences were 5'-GAACGUAGAUUAGCUUAUATT-3' and 5'-UAUAAGCUAAUCUACGUUCTG-3'.

#### Quantitative reverse transcription polymerase chain reaction (qRT-PCR)

Total RNA was extracted from cells using RNAiso reagent (TaKaRa, 9108). RNA (1 μg) was reverse-transcribed into cDNA using ReverTra Ace™ qPCR RT Master Mix (Toyobo, FSQ301), and qRT-PCR was performed using KAPA SYBR® FAST qPCR Master Mix (NIPPON Genetics Co. Ltd, KK4602) and a Roche LightCycler 480 system. Data were normalized to GAPDH levels.

#### Luciferase reporter assay

Cells were transfected with a Renilla luciferase reporter plasmid under the control of the IFN-β promoter (IFN-β-Luc), a Renilla luciferase reporter plasmid containing repeated IRF-binding sites (C1B-Luc), or a Renilla luciferase reporter plasmid containing NF-κB-binding sites (NF-κB-Luc), along with Firefly luciferase expression plasmid (control). Luminescence was measured using a Dual-Luciferase® Reporter Assay System (Promega, E1910).

#### Western blotting and Phos-Tag SDS-PAGE

Western blotting and Phos-Tag SDS-PAGE were performed as described previously.^47^

#### Immunoprecipitation/coimmunoprecipitation

Immunoprecipitation and immunoblot analysis were performed as previously described.^16^^,^^48^

#### Bacterial alkaline phosphatase (BAP) treatment

Alkaline phosphatase treatment as performed as described previously.^49^ Briefly, HEK293T cells were lysed with TNE buffer (50 mM Tris-HCl (pH 7.5), 150 mM NaCl, 1 mM EDTA, 1% NP-40) containing Phos-stop (Roche). After immunoprecipitation, the immunoprecipitates were incubated with 2.5 units of calf intestinal alkaline phosphatase (Takara) in BAP buffer (under rotation at 700 rpm for 1 h at 37°C). The resulting immunoprecipitates were then subjected to immunoblot analysis.

#### Mass spectrometry

HEK293T cells expressing His10-tagged human MAVS with or without active ASK kinases were lysed in 6 M guanidine-HCl, 100 mM HEPES-NaOH (pH8.0), and 2 mM DTT. After heating and sonication, the lysates were subjected to centrifugation (20,000 ×g) at 4°C for 15 min. The resultant supernatants were incubated at 4°C for 1 h in the presence of 5 μL of TALON resin (Clontech). The beads were washed four times with 6 M guanidine-HCl, 50 mM HEPES-NaOH (pH7.5), 150 mM NaCl, and 0.1% NP-40, and then twice with 50 mM Tris-HCl (pH8.0) and 1 M urea. Proteins on the beads were digested by adding 200 ng trypsin/Lys-C mix (Promega) followed by incubation overnight at 37°C. The digests were reduced, alkylated, acidified, and desalted with GL-Tip SDB (GL Sciences). The eluates were evaporated and dissolved in 0.1% trifluoroacetic acid and 3% acetonitrile. LC-MS/MS analysis of the resultant peptides was performed using an EASY-nLC 1200 UHPLC connected to a Q Exactive Plus mass spectrometer through a nanoelectrospray ion source (Thermo Fisher Scientific). The peptides were separated for 0–150 min on a C18 reverse-phase column (75-μm inner diameter × 150 mm; Nikkyo Technos) with a linear 4%–32% acetonitrile gradient, followed by an increase to 80% acetonitrile for 20 min and a final hold at 80% acetonitrile for 10 min. The mass spectrometer was operated in data-dependent acquisition mode with the top 10 MS/MS method. MS1 spectra were measured at a resolution of 70,000, an automatic gain control target of 1e6, and a mass range from 350–1500 *m/z*. HCD MS/MS spectra were triggered at a resolution of 17,500, an automatic gain control target of 5e4, an isolation window of 2.0 *m/z*, a maximum injection time of 60 ms, and a normalized collision energy of 27. Dynamic exclusion was set to 20 s. Raw data were analyzed directly against the SwissProt database (restricted to *Homo sapiens*) using Proteome Discoverer 2.5 (Thermo Fisher) and the Sequest HT search engine. The search parameters were as follows: (a) trypsin as an enzyme with up to two missed cleavages; (b) precursor mass tolerance of 10 ppm; (c) fragment mass tolerance of 0.02 Da; (d) carbamidomethylation of cysteine as a fixed modification; and (e) acetylation of the protein N-terminus, oxidation of methionine, and phosphorylation of serine, threonine, and tyrosine as variable modifications. Peptides and proteins were filtered at a false discovery rate (FDR) of 1% using the Percolator node and Protein FDR Validator node, respectively. Label-free quantification was performed based on intensities of precursor ions using the Precursor Ions Quantifier node. Normalization was performed such that the total sum of abundance values for each sample (over all peptides) was the same.

### Quantification and statistical analysis

For all bar graphs, data are expressed as the mean ± SEM. Prism 8 software (graphic software) was used for statistical analysis and to generate the graphs. The specific statistical tests used are stated in each figure legend. P < 0.05 was considered as statistically significant (∗p < 0.05, ∗∗p < 0.01, ∗∗∗p < 0.001; ∗∗∗∗p < 0.0001, NS, non-significant).

## Supplemental information

Document S1. Figures S1–S10

Table S1. Excel file related proteomic identification of MAVS phosphorylation sites
