# Supplementary material for: MAVS phosphorylation acts as a cellular stress sensor that modulates antiviral immunity
Source: iScience. 2025 Jul 31;28(9):113256. doi: 10.1016/j.isci.2025.113256 (PMC12362409; doi:10.1016/j.isci.2025.113256)
Supplement: Document S1. Figures S1–S10 [file mmc1.pdf]

iScience, Volume *28*

Supplemental information

MAVS phosphorylation acts as a cellular stress sensor that modulates antiviral immunity

Dongyi Zhao, Nao Morimoto, Riho Saito, Juri Yamada, Shuntaro Abe, Hidetaka Kosako, Yukiko Gotoh, and Tomohiko Okazaki

Supplemental Figures

Fig. S1

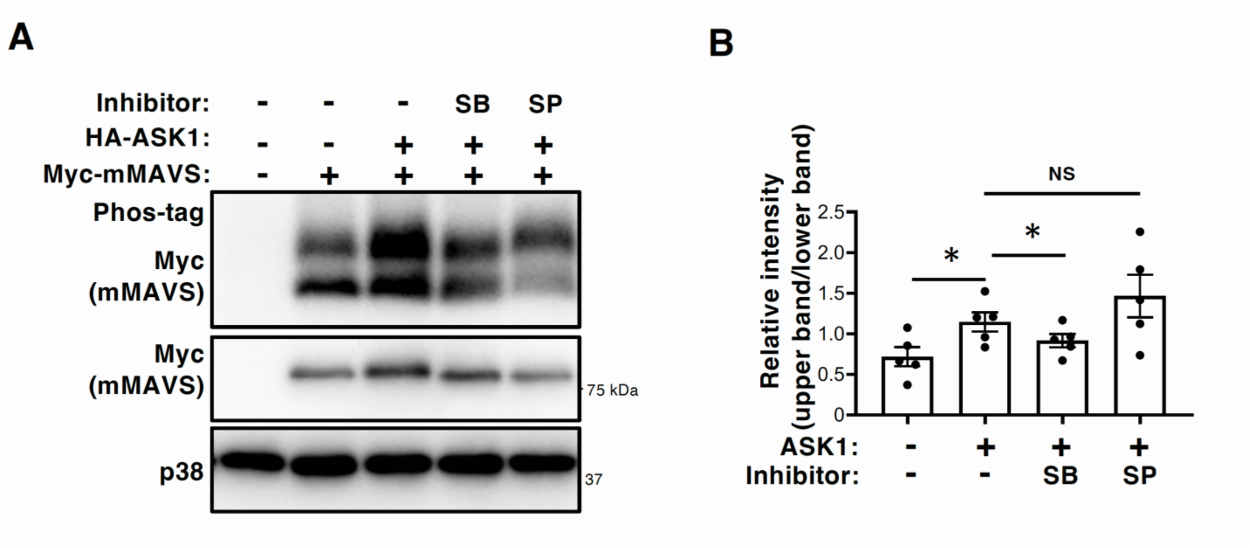

Fig. S1. ASK1-induced band shift of human MAVS is suppressed by the p38 inhibitor SB202190 but not by the JNK inhibitor SP600125.

(**A,B**) HEK293T cells transiently transfected with expression vectors for HA-tagged ASK1 and Myc-tagged mouse MAVS were treated with either the p38 inhibitor SB202190 (SB) or the JNK inhibitor SP600125 (SP), respectively, for 8 h. The cell lysates were then separated by SDS-PAGE in Phos-tag gels or normal gels, and then subjected to immunoblot analysis with antibodies specific for Myc or p38 (**A**). Results are representative of five independent experiments. Densitometry analysis of the upper band relative to the lower anti-Myc band in the immunoblot of Phos-tag gels (**B**). Data are presented as the mean ± SEM from five independent experiments (*$p<0.05$, non-significant (NS), one-way ANOVA with Dunnett's multiple comparison test).

Fig.S2

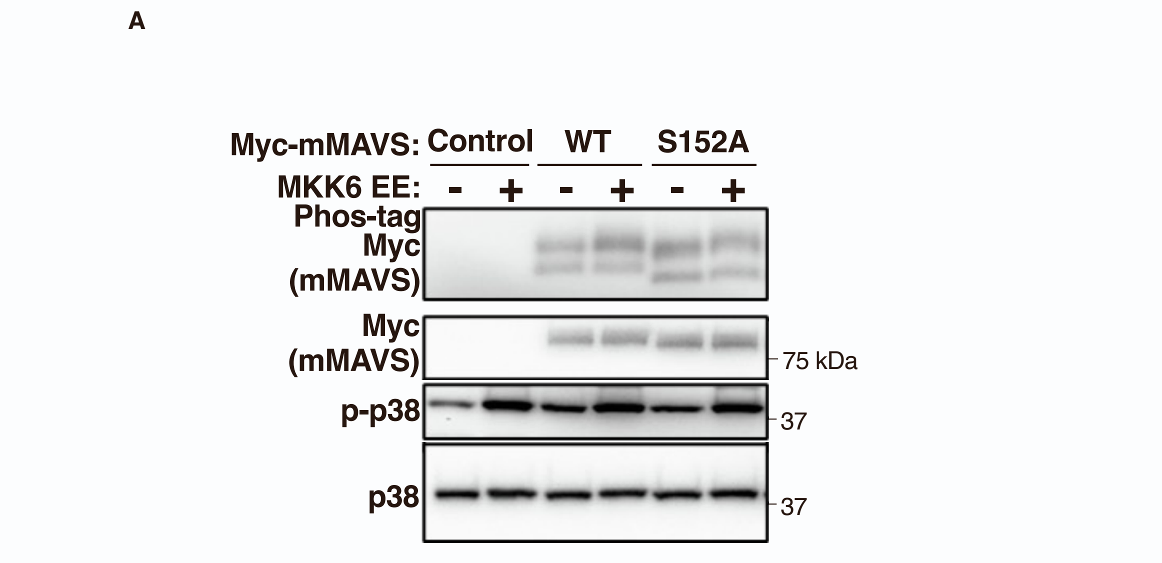

Fig. S2. Activation of p38 induces a shift in the band representing the MAVS S152A mutant.

HEK293T cells transiently transfected for 20 h with expression vectors for MKK6 EE and Myc-tagged WT mMAVS or Myc-tagged mMAVS S152A were lysed, and proteins separated by SDS-PAGE in Phos-tag gels or normal gels. This was followed by immunoblot analysis with antibodies specific for Myc, p-p38, or p38. Results are representative of three independent experiments.

Fig.S3

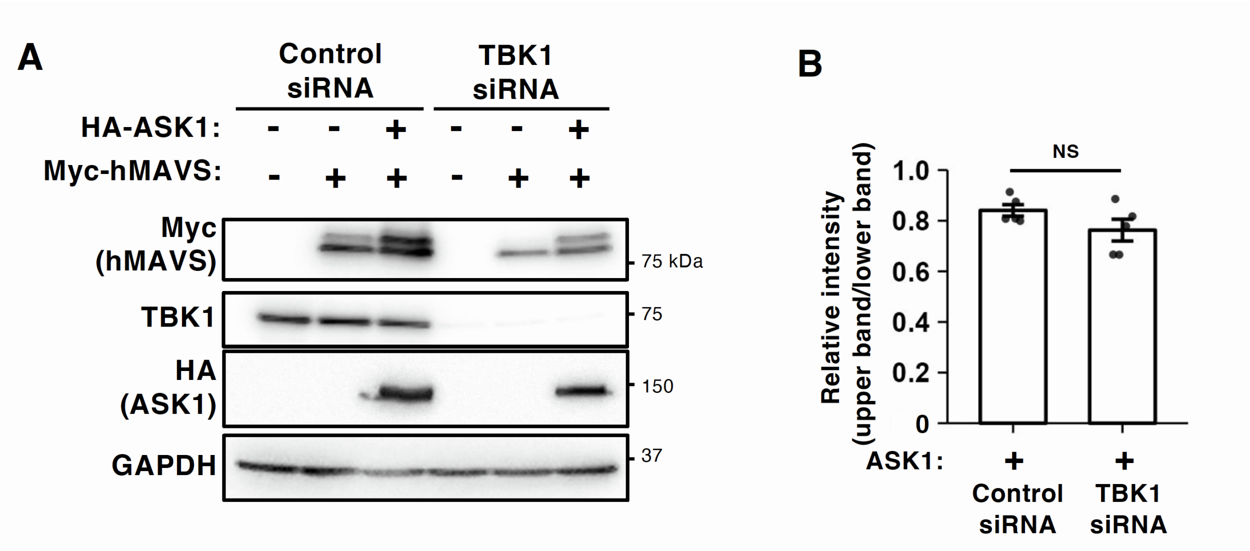

Fig. S3. Effect of TBK1 knockdown on ASK1-induced MAVS phosphorylation.

(A,B) 293T cells transfected with TBK1 or control siRNAs for 4 days were transiently transfected with expression vectors for Myc-tagged human MAVS and HA-tagged ASK1 for 20 h. The cell lysates were separated by SDS-PAGE, and then subjected to immunoblot analysis with antibodies specific for Myc, TBK1, HA or GAPDH (**A**). Results are representative of five independent experiments. Densitometry analysis of the upper band relative to the lower anti-Myc band in the immunoblot under conditions of ASK1 overexpression (**B**). Data are presented as the mean ± SEM from five independent experiments (*$p<0.05$, non-significant (NS), two-tailed Student's t-test).

Fig. S4

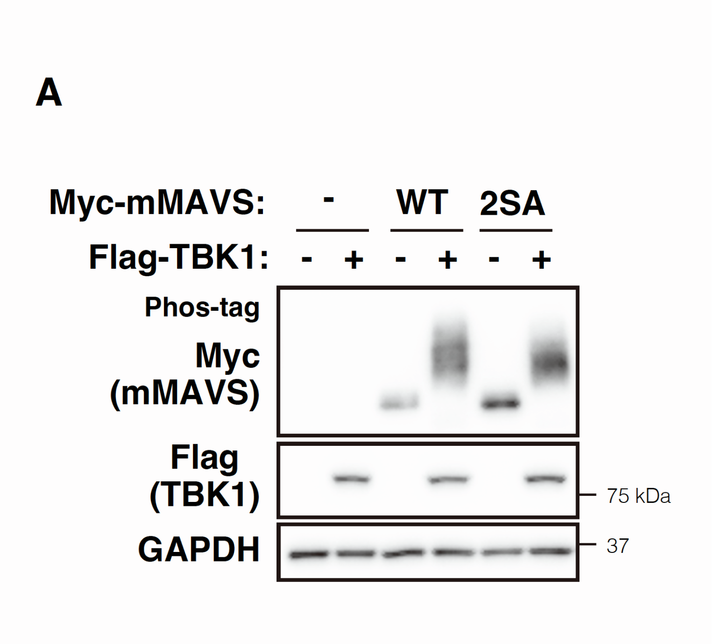

Fig. S4. Effect of 2SA mutation on TBK1-induced phosphorylation-associated band shift of MAVS.

(**A**) HEK293T cells transiently transfected for 20 h with expression vectors for Flag-tagged TBK1 and Myc-tagged WT mouse MAVS (mMAVS), Myc-tagged mMAVS S186A S220A (2SA). The cell lysates were separated by SDS-PAGE in Phos-tag gels or normal gel and then subjected to immunoblot analysis with antibodies specific for Myc, Flag, or GAPDH. Results are representative of three independent experiments.

Fig. S5

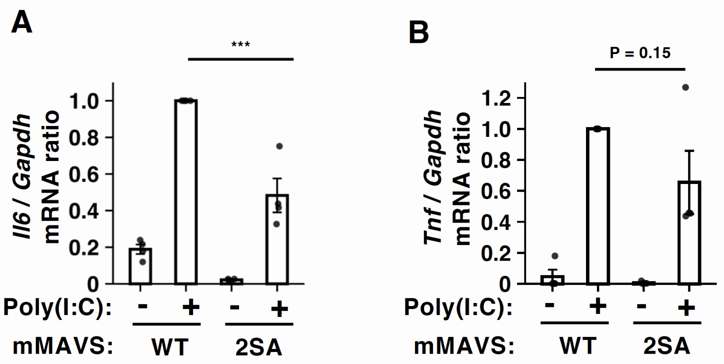

Fig. S5. MAVS phosphorylation sites are important for expression of proinflammatory cytokines in response to poly(I:C) stimulation.

(**A,B**) MAVS KO MEFs reconstituted with MAVS WT or the 2SA mutant were transfected with 0.25 μg/mL poly(I:C) for 3 h. Next, levels of *Il6* (**A**) or *Tnf* (**B**) mRNA were measured by reverse transcription and quantitative polymerase chain reaction (RT-qPCR) analysis. Data are expressed as the mean ± SEM of four independent experiments (***p<0.001, one-way ANOVA with Tukey' s multiple comparison test).

Fig. S6

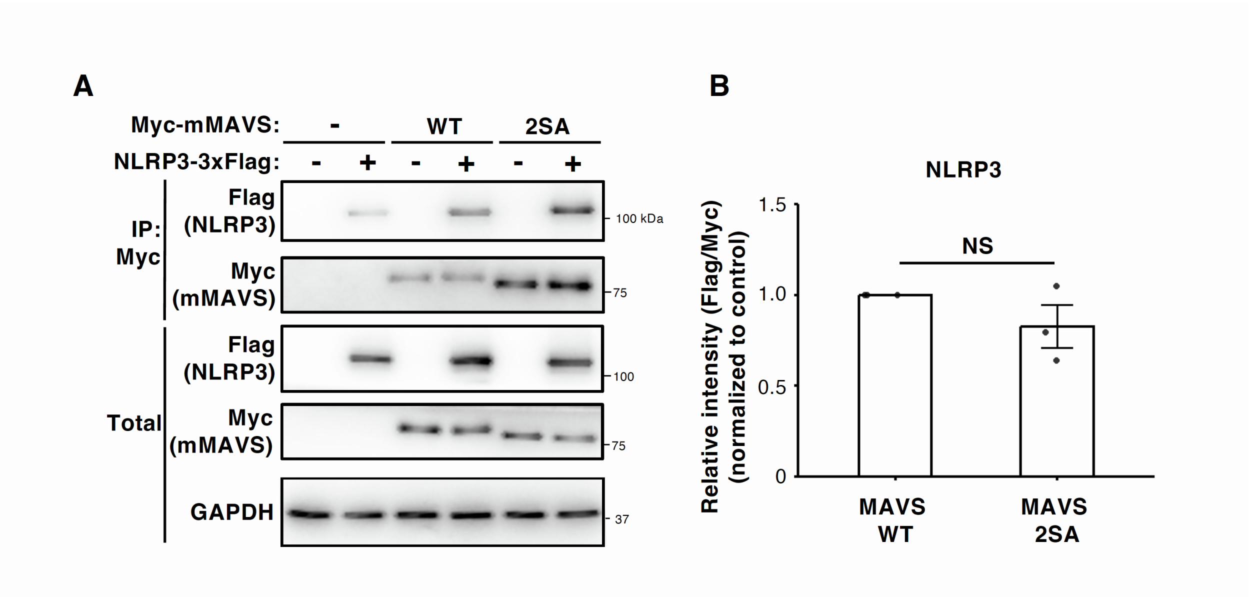

Fig. S6. Effect of the MAVS 2SA mutation on its interaction with NLRP3.

(**A,B**) HEK293T cells transiently transfected for 20 h with expression vectors for Flag-tagged NLRP3 and Myc-tagged WT mMAVS, or Myc-tagged mMAVS S186A/S220A (2SA) were subjected to IP with an anti-Flag antibody, and the resulting precipitates were then subjected to immunoblot analysis, together with the original cell lysates (Total), with antibodies specific for Flag, Myc (**A**). Results are representative of three independent experiments. Densitometry analysis of the Myc band relative to the FLAG band in the immunoprecipitate (**B**). Data are expressed as the mean ± SEM from three independent experiments (non-significant (NS), one sample t-test).

Fig. S7

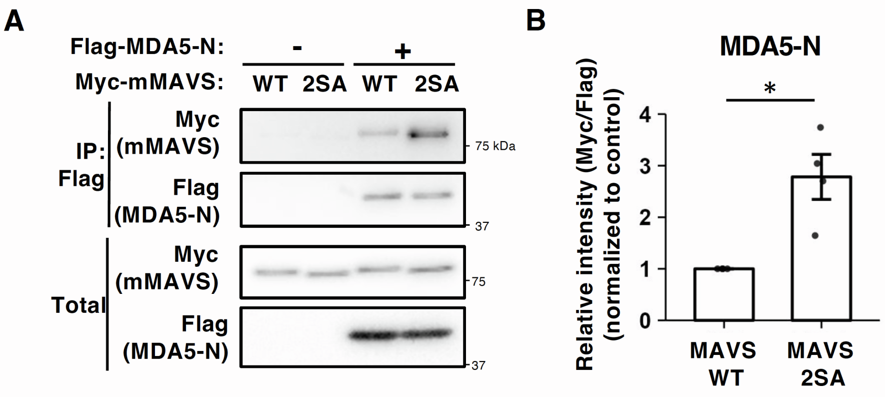

Fig. S7. Effect of the MAVS 2SA mutation on its interaction with MDA5.

(**A,B**) HEK293T cells transiently transfected for 20 h with expression vectors for Flag-tagged constitutively active N-terminal CARD domains of MDA5 (MDA5-N) and Myc-tagged WT mMAVS, or Myc-tagged mMAVS S186A/S220A (2SA) were subjected to IP with an anti-Flag antibody, and the resulting precipitates were then subjected to immunoblot analysis, together with the original cell lysates (Total), with antibodies specific for Flag, Myc (**A**). Results are representative of four independent experiments. Densitometry analysis of the Myc band relative to the FLAG band in the immunoprecipitate (**B**). Data are expressed as the mean ± SEM from four independent experiments (*p<0.05, one sample t-test).

Fig. S8

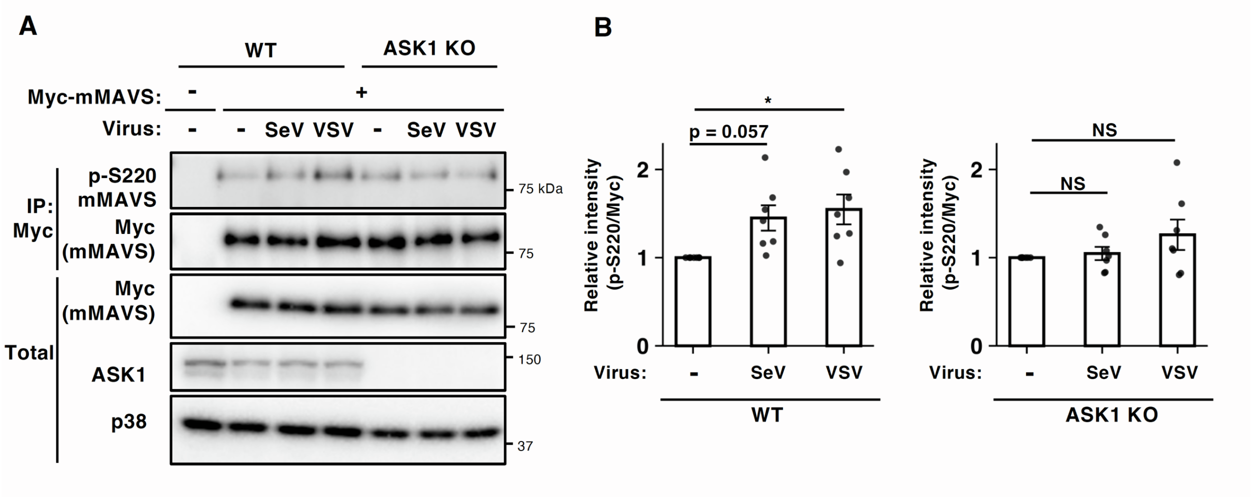

Fig. S8. ASK1 is required for full induction of MAVS S220 phosphorylation upon viral infection.

(**A, B**) HEK293T cells or ASK1 KO HEK293T cells transiently transfected for 20 h with expression vectors for Myc-tagged mouse MAVS (mMAVS) were infected with Sendai virus (SeV) or Vesicular Stomatitis Virus (VSV), and then subjected to immunoprecipitation (IP) with an anti-Myc antibody. The resulting precipitates were then subjected to immunoblot analysis, together with the original cell lysate (Total), with antibodies specific for phospho-S220 MAVS, Myc, ASK1, or p38 (**A**). Results are representative of seven independent experiments. Densitometry analysis of the phospho-S220 MAVS band relative to the Myc band in the immunoprecipitated, with normalization to the uninfected control performed independently for WT and ASK1 KO (**B**). Data are expressed as the mean ± SEM from seven independent experiments (*$p<0.05$, non-significant (NS), one-way ANOVA with Tuley's multiple comparisons test).

Fig.S9

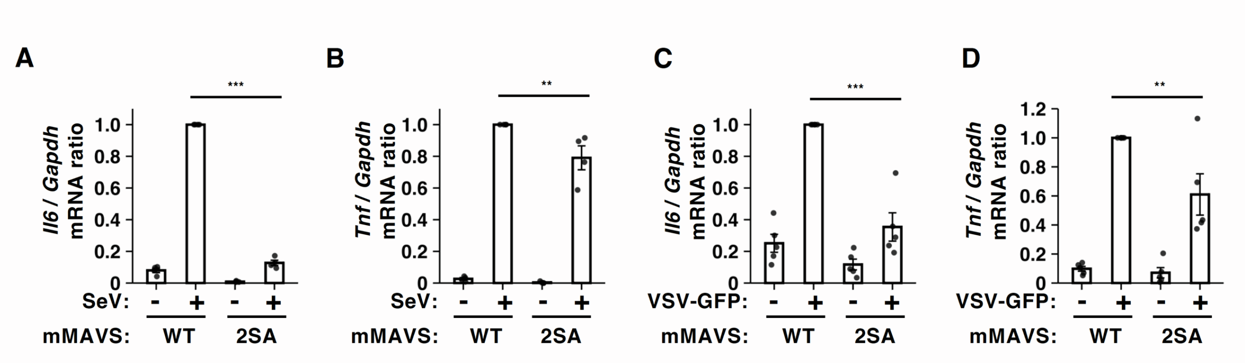

Fig. S9. MAVS phosphorylation sites are important for expression of proinflammatory cytokines in response to virus infection.

(**A,B**) MAVS KO MEFs reconstituted with MAVS WT or the 2SA mutant were infected with SeV or VSV-GFP for 3 h. Next, levels of *Il6* (**A, C**) or *Tnf* (**B, D**) mRNA were measured by reverse transcription and quantitative polymerase chain reaction (RT-qPCR) analysis. Data are expressed as the mean ± SEM of four (**A, B)** or five (**C, D**) independent experiments (**p<0.01,***p<0.001, one-way ANOVA with Tukey’ s multiple comparison test).

Fig.S10

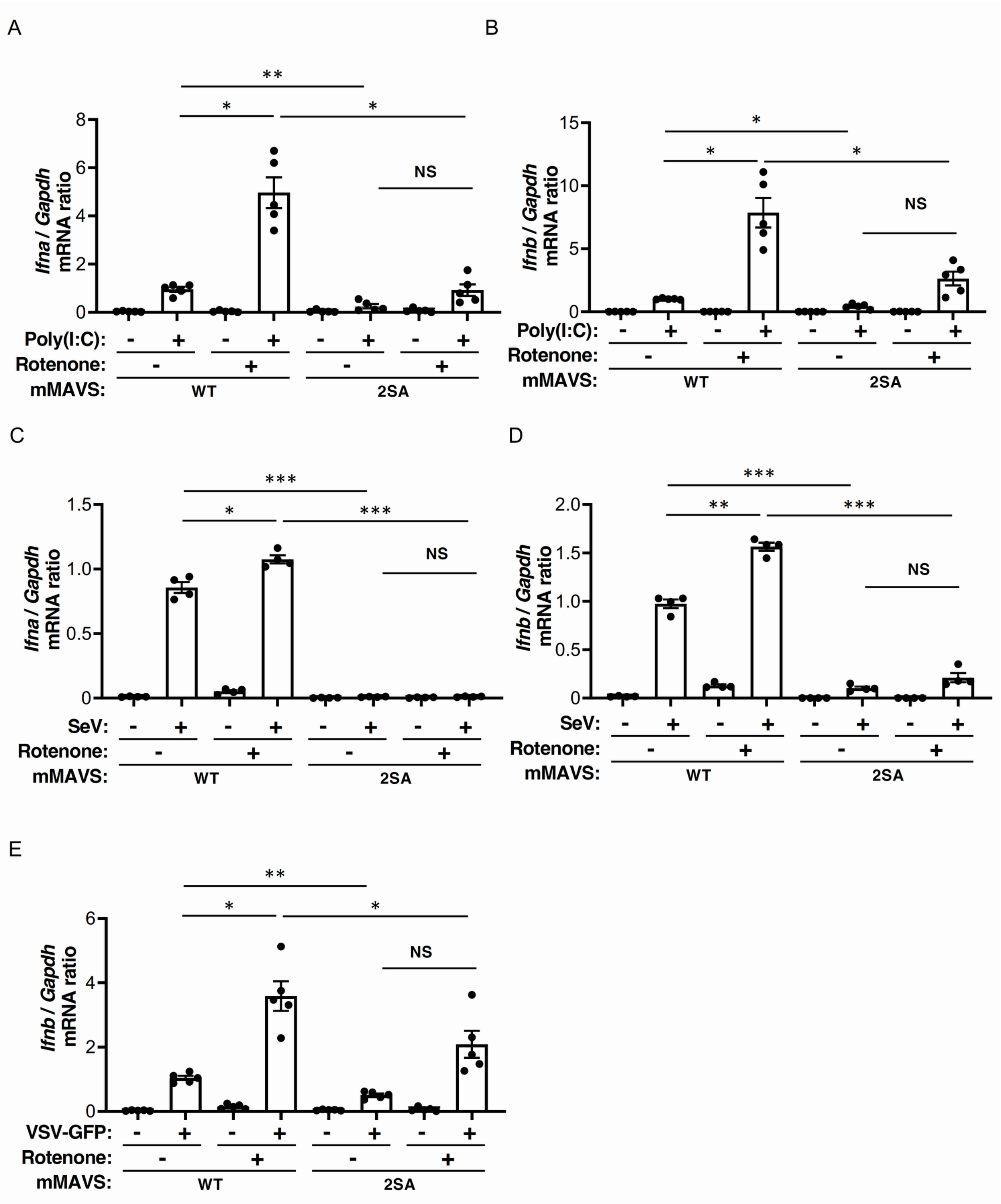

A
B
C
D
E
Ifna / Gapdh mRNA ratio
Ifnb / Gapdh mRNA ratio
Poly(I:C):
SeV:
VSV-GFP:
Rotenone:
mMAVS:
WT
2SA
NS

Fig. S10. Oxidative stress increases expression of type I IFNs via phosphorylation of MAVS.

(**A, B**) MAVS KO MEFs reconstituted with MAVS WT or the 2SA mutant were treated for 24 h with 1 μM of Rotenone and then transfected for 3 h with 0.25 μg/mL of poly(I:C). Cells were then subjected to reverse transcription and quantitative polymerase chain reaction (RT-qPCR) analysis to detect *Ifna* (**A**) or *Ifnb1* (**B**) mRNA. Data are expressed as the means ± SEM of five independent experiments (*p<0.05, **p<0.01, non-significant (NS), one-way ANOVA with Tukey's multiple comparisons test).

(**C, D**) MAVS KO MEFs reconstituted with MAVS WT or the 2SA mutant and treated with 1 μM of Rotenone for 24 h were infected with SeV for 3 h, before being subjected to reverse transcription and quantitative polymerase chain reaction (RT-qPCR) analysis to detect *Ifna* (**C**) or *Ifnb1* (**D**) mRNA. Data are expressed as the mean ± SEM of four independent experiments (*p<0.05, **p<0.01, ***p<0.001, non-significant (NS), one-way ANOVA with Tukey's multiple comparisons test).

(E) MAVS KO MEFs were reconstituted with MAVS WT or the 2SA mutant, treated with 1 μM of Rotenone for 24 h, and then infected with VSV-GFP for 3 h. Cells were then subjected to reverse transcription and quantitative polymerase chain reaction (RT-qPCR) analysis to detect *Ifnb1* mRNA. Data are expressed as the mean ± SEM of five independent experiments (*p<0.05, **p<0.01, non-significant (NS), one-way ANOVA with Tukey's multiple comparisons test).

Supplemental Table

Table S1. Excel file related proteomic identification of MAVS phosphorylation sites.

This table is provided as a separate Excel file.
